# Prediction of the Chloride Resistance of Concrete Modified with High Calcium Fly Ash Using Machine Learning

**DOI:** 10.3390/ma8125483

**Published:** 2015-12-11

**Authors:** Michał Marks, Michał A. Glinicki, Karolina Gibas

**Affiliations:** 1 Research and Academic Computer Network,Wawozowa 18, Warsaw 02-796, Poland; 2 Institute of Fundamental Technological Research, Polish Academy of Sciences, Pawinskiego 5B, Warsaw 02-106, Poland; mglinic@ippt.gov.pl (M.A.G.); kgibas@ippt.gov.pl (K.G.)

**Keywords:** chloride penetration, concrete, durability, high calcium fly ash, machine learning

## Abstract

The aim of the study was to generate rules for the prediction of the chloride resistance of concrete modified with high calcium fly ash using machine learning methods. The rapid chloride permeability test, according to the Nordtest Method Build 492, was used for determining the chloride ions’ penetration in concrete containing high calcium fly ash (HCFA) for partial replacement of Portland cement. The results of the performed tests were used as the training set to generate rules describing the relation between material composition and the chloride resistance. Multiple methods for rule generation were applied and compared. The rules generated by algorithm J48 from the Weka workbench provided the means for adequate classification of plain concretes and concretes modified with high calcium fly ash as materials of good, acceptable or unacceptable resistance to chloride penetration.

## 1. Introduction

The increased use of high calcium fly ash (HCFA) for partial replacement of Portland cement in concrete could result in a number of environmental benefits (reduced consumption of cement clinker, reduced CO${}_{2}$ emissions during cement production, saving natural resources, reduced landfill space and storage costs). The resources of high calcium fly ash are large, it is produced as a by-product of power generation in brown coal burning plants. However, this type of ash is usually characterized by low silica content, a high content of free lime and an increased content of sulfur compounds. It could be used in concrete following the requirements of ASTM (American Society for Testing and Materials) C618 Class C, but in Europe, it does not meet the requirements defined in standard EN 450-1. At present, HCFA is not in common use in European countries in spite of positive examples of its suitability provided by Greek and Turkish researchers. It was shown [1] that in the case of cement replacement with HCFA, the compressive strength of concrete was increased if the content of active silica in the fly ash was higher than that in the cement. Similar results were obtained earlier by Naik, *et al.* [2]: partial replacement of cement by fine-grained HCFA resulted in the same or better compressive strength of concrete; the results for drying shrinkage were also positive. The optimization of fineness coupled with the adjustment of water content were found as the key parameters of the effective utilization of high calcium fly ashes for strength maximization of cement mortars [3]. The application of HCFA as a partial cement replacement in mortar beams stimulated self-healing of cracks and particularly of microcracks [4]. It was also found that concrete specimens incorporating HCFA exposed to long-term chloride ponding experiments exhibited significantly lower total chloride content for all depths from the surface [5]. The key factors for the adequate performance of HCFA in concrete seem to be both the composition and the gradation of fly ash.

The assessment of concrete resistance to chloride ingress is fundamental for the durability of reinforced concrete structures exposed to deicing salt and the marine environment [6]. Numerous papers on chloride penetration resistance of concrete modified with standard siliceous fly ash were recently reviewed in [7]. The addition of fly ash is generally found (and confirmed in [8]) to reduce chloride permeability and also to increase the chloride binding capacity of concrete. Despite lower chloride threshold values, the addition of fly ash was found to provide better corrosion protection to steel reinforcements. There is a need to extend such a study to include high calcium fly ash. For rational use of HCFA in structural concrete, there is also a need to propose tools for the prediction of the chloride penetration resistance of concrete.

The prediction of the engineering properties of composite materials is usually based on experimental test results with a reference to the observed material microstructure. The relevant material characteristics can be extracted from an experimental dataset using various artificial intelligence methods, developed for the last two decades for various engineering applications [9,10]. Artificial neural networks were successfully applied for the prediction of the compressive strength of concrete containing silica fume [11] or coal ash [12]. Moreover, the application of neural networks and optimization technologies created the possibility to search for the optimum mixture of concrete: the mixture with the lowest cost and required performance, such as strength and slump [13]. Machine learning methods were also tested on the classification of concrete modified by fluidized bed fly ash as materials of adequate resistance to chloride penetration [14] and resistance to surface scaling [15]. The application of machine learning for the prediction of the scaling resistance of concrete modified with high calcium fly ash is described in [16]. The authors of [17,18] proposed to combine artificial neural networks and machine learning methods in one system to estimate and predict various properties of concrete materials.

The aim of this study is to generate rules using a machine learning algorithm to evaluate the chloride resistance of concrete modified with high calcium fly ash. The rules are generated using selected attributes from a database created by storing the experimental results of the chloride migration coefficient determined for three concrete series.

## 2. Composition of Concrete Mixes and Test Results of the Chloride Migration Coefficient

The chloride migration coefficient in concrete specimens with different contents of high calcium fly ash was experimentally measured. Concrete mixes were prepared with high calcium fly ash used for replacement of 15% or 30% of the cement mass. Experimental tests were performed on several mixes. For concrete manufacturing, two types of Portland cement, CEM I 42.5R (with 10% C${}_{3}$A content) or CEM I 42.5 HSR NA (with 2% C${}_{3}$A content), siliceous sand fraction 0÷2 mm and amphibolite as a coarse aggregate (two fractions 2÷8 mm and 8÷16 mm) were used. The following admixtures were used: a high range water reducer (based on polycarboxylate ethers) and a plasticizer (lignosufonate). Because of the expected variability of ash properties, three lots of high calcium fly ash were tested from different deliveries from the power plant, namely S1, 16 March 2010, S2, 19 May 2010, and S3, 28 June 2010. The chemical composition of HCFA is given in Table 1. For HCFA beneficiation, a grinding process was applied during 10–28 minutes in a ball mill. The physical properties of ash before and after grinding are given in Table 2 [19]. HCFA was used as an additive to concrete mix in unprocessed form (as collected) and after grinding.

The Nordtest Method Build 492—Non-Steady State Migration Test [20] was used to determine the chloride migration coefficient. The principle of the test is to subject the concrete specimen to external electrical potential applied across it and to force chloride ions to migrate into the concrete. The specimens are then split open and sprayed with silver nitrate solution, which reacts to give white insoluble silver chloride on contact with chloride ions. This provides a possibility to measure the depth to which a sample has been penetrated. The non-steady-state migration coefficient, $D_{nssm}$, is determined on the basis of Fick’s second law. This coefficient is dependent on the voltage magnitude, the temperature of the anolyte measured at the beginning and the end of test and the depth of chloride ions’ penetration. The criteria for evaluating the resistance of concrete against chloride penetration proposed by L. Tang [21] are shown in Table 3.

**Table 1:** The chemical composition of high calcium fly ashes from Bełchatów power plant in Poland, determined using the XRF (X-ray fluorescence) method. Fly ash sampling date and bath designation [19].

Component	Fly Ash Sampling Date and Batch Designation		
	16.03.2010	19.05.2010	28.06.2010
	S1	S2	S3
LOI	2.56%	3.43%	1.85%
SiO${}_{2}$	33.62%	35.41%	40.17%
Al${}_{2}$O${}_{3}$	19.27%	21.86%	24.02%
Fe${}_{2}$O${}_{3}$	5.39%	6.11%	5.93%
CaO	31.32%	25.58%	22.37%
MgO	1.85%	1.49%	1.27%
SO${}_{3}$	4.50%	4.22%	3.07%
K${}_{2}$O	0.11%	0.13%	0.20%
Na${}_{2}$O	0.31%	0.16%	0.15%
P${}_{2}$O${}_{5}$	0.17%	0.16%	0.33%
TiO${}_{2}$	1.21%	1.22%	1.01%
Mn${}_{2}$O${}_{3}$	0.07%	0.06%	0.06%
SrO	0.20%	0.17%	0.16%
ZnO	0.02%	0.02%	0.02%
CaO${}_{free}$	2.87%	1.24%	1.46%

**Table 2:** Physical properties of high calcium fly ashes before and after processing [19].

Batch	Fly Ash Designation	Density (g/cm${}^{3}$)	Fineness: The Residue on Sieve 45 *μ*m (%)	Specific Surface by Blaine (cm${}^{2}$/g)
S1	S1: unprocessed	2.62	38.0	2860
	S1${}_{10}$: ground 10 min	2.77	23.0	3500
	S1${}_{28}$: ground 28 min	2.75	10.5	3870
S2	S2: unprocessed	2.58	35.4	4400
	S2${}_{15}$: ground 15 min	2.70	13.3	6510
S3	S3: unprocessed	2.64	55.6	1900
	S3${}_{20}$: ground 20 min	2.71	20.0	4060

**Table 3:** Criteria for the classification of the concrete resistance to chloride ions’ penetration [21].

Chloride Migration Coefficient $D_{nssm}$	Resistance to Chloride Penetration
<2 × 10${}^{-12}$ m^2^/s	Very good
2-8 × 10${}^{-12}$ m^2^/s	Good
8-16 × 10${}^{-12}$ m^2^/s	Acceptable
>16 × 10${}^{-12}$ m^2^/s	Unacceptable

Experimental tests revealed a decrease of the chloride migration coefficient with the increase in the HCFA amount added to the mix. The most significant reduction of $D_{nssm}$ by 36%–75% and 54%–89% after 28 and 90 days of curing, respectively, was obtained when using ground HCFA to substitute 30% of binder mass. With a such reduction of $D_{nssm}$, the level of chloride resistance changed from acceptable to good or from unacceptable to acceptable, [22]. For a few mixes prepared with a water-to-binder ratio of 0.60, a change of $D_{nssm}$ did not increase the level of chloride penetration resistance. Sieving through a 0.125-mm mesh size sieve was found to improve HCFA performance: it significantly reduced the value of $D_{nssm}$, which was most evident after 90 days of curing. No clear relationship could be found between $D_{nssm}$ and the water-to-binder ratio or the compressive strength of concrete.

The resistance against chloride ingress of concrete containing low calcium fly ash was previously tested by Baert, *et al.* [23], and at 28 days, the chloride migration coefficient was increased with increasing fly ash content. However at later ages (3, 6 or 12 months), due to the pozzolanic reaction, the $D_{nssm}$ coefficient was lower for all concrete mixes with siliceous fly ash. The effects of blast furnace slag on the chloride migration coefficient summarized by Gjorv [6] were clearly favorable, even at the age of 14 days. After 28 days of water curing, the increasing amounts of slag up to 80% replacement resulted in the reduced apparent chloride diffusion coefficient from 11 × $10^{-12}$ down to 2 × $10^{-12}$ m/s${}^{2}$. The comparison with the obtained results on HCFA in concrete reveals almost comparable efficiency as blast furnace slag. This could be attributed to both pozzolanic and hydraulic activity of HCFA. The hydraulic properties of these fly ashes should be related to reactive aluminate phases and their hydration and also to the formation of ettringite in the initial phase of hydration [24]. A high hydraulic and pozzolanic activity index after a prolonged hydration and hardening process is connected with hydraulic phases, mainly belite and gehlenite, as well as with the reactivity of the glassy phase. The complexity of the phenomena involved in chloride ion penetration in concrete containing such a mineral addition of pozzolanic and hydraulic activity justifies an application of machine learning techniques to reveal the possible governing rules. 

In Table 4, the database containing data on the composition of the concrete mixes, the specific surface of fly ash obtained by the Blaine method and the chloride migration coefficient determined after 28 days of curing is presented. The estimation of the concrete resistance to chloride penetration, based on the values of the diffusion coefficients according to the criterion presented in Table 3, is placed in the last column of Table 4. 

The permeability of concrete is known to be dependent largely on the water-to-cement ratio, (w/c). However the definition of w/c is not unambiguous when using supplementary cementitious materials. Following the EN 206 standard, the effect of active mineral additions on w/c is quantified using the *k*-efficiency factor: the content of the additive (*a*) is multiplied with a *k*-value, and the water to cement ratio (w/c) is replaced by ${\left(w/c\right)}_{eq}=w/\left(c+k\cdot a\right)$. The efficiency *k* factor approach is adequate to address the mix design for compressive strength when using the additives of the established efficiency. Even in such a case, like siliceous fly ash, the efficiency factors are not the same for durability performance and for the compressive strength [25]. The compiled fly ash efficiency data [6,26] revealed a much higher efficiency coefficient *k* in relation to the compressive strength than the value given in EN 206, even reaching the value of two in relation to the resistance to chloride attack. For nonstandard fly ashes and coal combustion products from so-called clean coal technology, the efficiency factors are not established [27]. Therefore, it is not possible to describe all of the effects of the nonstandard fly ashes, including HCFA, on concrete performance when exposed to various environmental factors with only one efficiency coefficient. In order to avoid an unambiguous (w/c) definition, the content of water in the mix is used as a descriptor in the machine learning database. 

**Table 4:** The database of the composition of concrete mixes and the properties of hardened concretes.

Concrete Mix	Content (kg/m^3^)											Specific Surface of Fly Ash (cm^2^/g)	Chloride Migration Coefficient ( × 10^−12^ m^2^/s)	Category of Resistance to Chloride Penetration
	Cement CEM I 42.5		High Calcium Fly Ash							Aggregate	Water			
	10% C_3_A	2% C_3_A												
mix	C1	C2	S1	S1_10_	S1_28_	S2	S2_15_	S3	S3_20_	K0_16_	w	surf	D_*nssm*_	resistance
R_38	359	0	0	0	0	0	0	0	0	1945	156	0	10.13	unacceptable
R_39	305	0	137	0	0	0	0	0	0	1848	153	2860	7.88	good
R_41	250	0	268	0	0	0	0	0	0	1741	152	2860	3.76	good
R_42	323	0	0	0	0	0	0	0	0	1938	174	0	23.73	unacceptable
R_43	272	0	120	0	0	0	0	0	0	1837	169	2860	12.36	unacceptable
R_44	226	0	241	0	0	0	0	0	0	1768	169	2860	8.10	unacceptable
R_47	310	0	0	139	0	0	0	0	0	1892	140	3500	5.44	good
R_48	257	0	0	275	0	0	0	0	0	1802	142	3500	3.42	good
R_49	275	0	0	121	0	0	0	0	0	1872	160	3500	17.79	unacceptable
R_50	228	0	0	244	0	0	0	0	0	1800	159	3500	10.37	unacceptable
R_51	306	0	0	0	137	0	0	0	>0	1852	153	3870	6.37	good
R_52	255	0	>0	0	273	0	0	0	>0	1780	153	3870	3.85	good
R_53	277	0	0	0	122	0	0	0	0	1871	175	3870	12.22	unacceptable
R_54	228	0	0	0	244	0	0	0	0	1784	173	3870	5.52	good
R_75	0	366	0	0	0	0	0	0	0	1997	143	0	11.96	unacceptable
R_76	0	312	140	0	0	0	0	0	0	1901	142	2860	6.34	good
R_77	0	251	270	0	0	0	0	0	0	1765	140	2860	4.04	good
R_78	0	328	0	0	0	0	0	0	0	1982	165	0	21.91	unacceptable
R_79	0	278	123	0	0	0	0	0	0	1894	159	2860	10.30	unacceptable
R_80	0	226	242	0	0	0	0	0	0	1790	157	2860	7.88	good
R_81	0	304	0	0	0	136	0	0	0	1861	133	4400	5.04	good
R_82	0	277	0	0	0	122	0	0	0	1889	158	4400	7.76	good
R_116	340	0	0	0	0	0	0	0	0	1841	170	0	20.79	unacceptable
R_125	296	0	0	0	0	0	0	75	0	1836	174	1900	8.17	unacceptable
R_118	237	0	0	0	0	0	0	145	0	1767	172	1900	10.95	unacceptable
R_117	295	0	0	0	0	0	0	0	74	1826	174	4060	12.00	acceptable
R_119	239	0	0	0	0	0	0	0	147	1781	171	4060	5.17	good
R_107	308	0	0	0	0	0	0	0	0	1846	186	0	26.00	unacceptable
R_102	265	0	0	0	0	0	0	67	0	1834	189	1900	22.80	unacceptable
R_103	218	0	0	0	0	0	0	134	0	1814	189	1900	20.86	unacceptable
R_105	265	0	0	0	0	0	0	0	67	1839	189	4060	12.10	acceptable
R_104	219	0	0	0	0	0	0	0	135	1820	190	4060	7.59	good
R_120	0	343	0	0	0	0	0	0	0	1862	172	0	23.09	unacceptable
R_122	0	239	0	0	0	0	0	146	0	1779	171	1900	21.85	unacceptable
R_121	0	295	0	0	0	0	0	0	74	1824	173	4060	19.61	unacceptable
R_123	0	240	0	0	0	0	0	0	147	1786	171	4060	17.65	unacceptable
R_106	0	312	0	0	0	0	0	0	0	1869	189	0	28.50	unacceptable
R_111	0	265	0	0	0	0	0	67	0	1836	187	1900	31.63	unacceptable
R_112	0	222	0	0	0	0	0	136	0	1840	191	1900	27.44	unacceptable
R_110	0	265	0	0	0	0	0	0	67	1840	187	4060	25.42	unacceptable
R_108	0	223	0	0	0	0	0	0	137	1852	192	4060	23.04	unacceptable
A_0	350	0	0	0	0	0	0	0	0	1890	158	0	14.38	acceptable
A_15	298	0	133	0	0	0	0	0	0	1800	158	2860	7.91	good
B_15	298	0	0	133	0	0	0	0	0	1800	158	3500	6.39	good
C_15	298	0	0	0	133	0	0	0	0	1800	158	3870	5.52	good
A_30	245	0	263	0	0	0	0	0	0	1710	158	2860	5.43	good
B_30	245	0	0	263	0	0	0	0	0	1710	158	3500	1.63	very good
C_30	245	0	0	0	263	0	0	0	0	1710	158	3870	1.52	very good
D_15	298	0	0	0	0	133	0	0	0	1800	158	4400	3.06	good
E_15	298	0	0	0	0	0	133	0	0	1800	158	6510	2.06	good
H_0	0	350	0	0	0	0	0	0	0	1880	175	0	37.04	unacceptable
H_15M	0	298	0	0	0	0	0	0	75	1847	175	4060	34.48	unacceptable
H_15S	0	298	0	0	0	0	0	75	0	1847	175	1900	33.03	unacceptable
H_30M	0	245	0	0	0	0	0	0	150	1813	175	4060	27.41	unacceptable
H_30S	0	245	0	0	0	0	0	150	0	1813	175	1900	27.59	unacceptable

The database presented in Table 4 is a general database, which can be transformed into a “working database” by column selection.

## 3. Machine Learning Methods Used in the Prediction of the Engineering Properties of Composite Materials

### 3.1. Introduction to Machine Learning

Determining the relationship between material composition and the chloride resistance of concrete is a difficult and time-consuming process, even in the case of a small dataset, as presented in Table 4. For the considered dataset, it requires simultaneous analysis of 12 attributes (columns) for over 50 examples (rows). This task can be done manually; however, using a computer system to support data exploration is much more efficient. The branch of artificial intelligence concerned with applying algorithms that let computers evolve patterns using empirical data is called machine learning.

The aim of machine learning is to automatically learn to recognize complex patterns and make intelligent decisions based on the dataset. By a dataset, we mean a collection of logically-related records: a database. Each record can be called an instance or example, and each one is characterized by the values of predetermined attributes. The difficulty lies in the fact that the set of all possible behaviors given all possible inputs is too large to be covered by the set of observed examples (training data). Hence, the learner must generalize from the given examples, so as to be able to produce a useful output in new cases.

Patterns recognition associated usually with classification is the most popular example of utilizing machine learning. However machine learning or, more general, statistical algorithms can support the knowledge discovery at different stages from outlier detection and attribute (features) selection to knowledge modeling and model validation.

### 3.2. Feature Selection

Feature selection, also known as attribute selection or feature reduction, is the technique of selecting a subset of relevant features for building robust learning models. By removing most irrelevant and redundant attributes from the data, feature selection helps improve the performance of learning models by: speeding up the learning process and alleviating the effect of the curse of dimensionality. Moreover, the irrelevant attributes degrade the performance of state-of-the-art decision tree and rule learners [28].

### 3.3. Classification

As was written earlier in Section 3.1, classification is the most common type of machine learning application. The goal of the classification process is to find a way of classifying unseen examples based on the knowledge extracted from the provided set of classified instances. Extracting the knowledge from the provided dataset requires the attribute set characterizing the example to be divided into two groups: the class attribute and the non-class attributes. For unseen instances, only non-class attributes are known; hence, the aim of data mining algorithms is to create such a knowledge model that allows predicting the example class membership based only on non-class attributes.

The knowledge model depends on the way the classifier is constructed, and it can be represented by classification rules (the algorithm AQ21 [29]), decision trees (e.g., algorithm C4.5, [30]) or many other representations. Regardless of the representation, both classification rules and decision trees algorithms create hypotheses.

In the considered problem, the chloride resistance of concrete (class attribute) depending on the material composition and some predictions of the concrete (non-class attributes) is searched. We concentrated on the most popular representative of decision tree classifiers, the J48 algorithm, the open-source implementation of the last publicly-available version of a C4.5 method developed by J. Ross Quinlan [30]. This algorithm was compared to selected algorithms available in Weka [28] in Section 4.2.

### 3.4. Classifier Evaluation

So as to evaluate the classifier, *i.e.*, to judge the hypotheses generated from the provided training set, we have to verify the classifier performance on the independent dataset, which is called the testing set. The classifier predicts the class of each instance from the test set; if it is correct, it is counted as a success; if not it, is an error. The measure of the overall performance of the classifier is the classification accuracy. This is the number of correct classifications of the instances from the test set divided by the total number of these instances, expressed as a percentage. The greater the classification accuracy, the better is the classifier.

In order to get a deeper understanding of which types of errors are the most frequent, the result obtained from a test set is often displayed as a two-dimensional confusion matrix with a row and a column for each class. Each matrix element shows the number of test examples, for which the actual class is the row and the predicted class is the column. Good results correspond to large numbers down the main diagonal and small, ideally zero, for the elements off the diagonal. The sum of the numbers down the main diagonal divided by the total number of test examples determine the classification accuracy.

Let’s consider what can be done when the number of data for training and testing is limited. The simplest way to handle this situation is to reserve a certain number of examples for testing and to use the remainder for training. Of course, the selection should be done randomly. The main disadvantage of this simple method is that this random selection may not be representative. A more general way to mitigate any bias caused by the particular sample chosen for hold out is to repeat the whole process, training and testing, several times with different random samples. The random selection repeated many times can be treated as the basis of a statistical technique called cross-validation. In the *k*-fold cross-validation, the dataset *U* is split into *k* approximately equal portions ($U=E_{1}⋃...⋃E_{k}$) [31]. In each iteration *i*, the set $E_{i}$ is used for testing, and the remainder $U\backslash E_{i}$ is used for training. Overall classification accuracy is calculated as an average from the classification accuracy for each iteration.

When we have only one database consisting of a very small number of records, the estimation of classification accuracy (the measure of the overall performance of the classifier) can be done using the *n*-fold cross-validation, where *n* is the number of examples in the database. In this method, called leave-one-out cross-validation, each example in turn is left out, and the learning method is trained on all of the remaining examples. It is judged by its correctness on the remaining example, one or zero for success or failure, respectively. The results from *n* judgments, one for each member of the database, are averaged, and that average represents the classification accuracy [28].

## 4. Searching for the Rules Describing the Chloride Resistance of Concrete Modified with HCFA

### 4.1. Feature Selection

In Table 4, the dataset with 12 attributes is presented. It is clear that for database with a few dozens of instances, this number of attributes is too large. Some attributes can be eliminated, but it is important to eliminate the most irrelevant attributes.

Therefore, we decided to evaluate a subset of attributes using the best first and exhaustive approaches to feature selection. The best first method searches the space of attributes by greedy hill climbing augmented with backtracking facility. In both cases, the *CfsSubsetEvaluator*, provided by Weka, was used to assess the predictive ability of each attribute individually and the degree of redundancy among them, preferring sets of attributes that are highly correlated with the class, but have low inter-correlation. Both methods of searching (best first and exhaustive) resulted in selection of C1, S1${}_{28}$, w and surf attributes as a percent of tests, as presented in Table 5.

**Table 5:** Attribute selection cross-validation results.

Attribute	C1	C2	S1	S1${}_{10}$	S1${}_{28}$	S2	S2${}_{15}$	S3	S3${}_{20}$	K0${}_{16}$	w	surf
Best First	100%	0%	0%	0%	32%	0%	0%	0%	0%	0%	100%	100%
Exhaustive Search	98%	0%	0%	0%	32%	0%	0%	0%	0%	0%	100%	100%

Therefore, in order to generate rules describing the chloride resistance of concrete modified with high calcium fly ash, the subset of attributes (C1, cement content with 10 percent of C${}_{3}$A content (kg/m${}^{3}$), S1${}_{28}$, high calcium fly ash ground 28 minutes content (kg/m${}^{3}$), w, water content (kg/m${}^{3}$), surf, specific surface of fly ash obtained by the Blaine method (cm${}^{2}$/g), and resistance, concrete resistance to chloride penetration (acceptable, good, unacceptable)) from the database (Table 4) is used. The shrunken database containing 56 records, each one described by four numerical and one nominal attributes, is presented in Table 6. The last attribute, resistance, denotes a class and can take one of three values (good, acceptable or unacceptable). Since the class “very good” representation is not sufficient (only two examples), we decided to assign them to the “good” class, which now covers 22 examples.

**Table 6:** The database.

Number	C1	S1${}_{28}$	w	surf	resistance
1	359	0	156	0	acceptable
2	305	0	153	2860	good
3	250	0	152	2860	good
4	323	0	174	0	unacceptable
5	272	0	169	2860	acceptable
6	226	0	169	2860	acceptable
7	310	0	140	3500	good
8	257	0	142	3500	good
9	275	0	160	3500	unacceptable
10	228	0	159	3500	acceptable
11	306	137	153	3870	good
12	255	273	153	3870	good
13	277	122	175	3870	acceptable
14	228	244	173	3870	good
15	0	0	143	0	acceptable
16	0	0	142	2860	good
17	0	0	140	2860	good
18	0	0	165	0	unacceptable
19	0	0	159	2860	acceptable
20	0	0	157	2860	good
21	0	0	133	4400	good
22	0	0	158	4400	good
23	340	0	170	0	unacceptable
24	296	0	174	1900	acceptable
25	237	0	172	1900	acceptable
26	295	0	174	4060	acceptable
27	239	0	171	4060	good
28	308	0	186	0	unacceptable
29	265	0	189	1900	unacceptable
30	218	0	189	1900	unacceptable
31	265	0	189	4060	acceptable
32	219	0	190	4060	good
33	0	0	172	0	unacceptable
34	0	0	170	1900	unacceptable
35	0	0	171	1900	unacceptable
36	0	0	173	4060	unacceptable
37	0	0	171	4060	unacceptable
38	0	0	189	0	unacceptable
39	0	0	187	1900	unacceptable
40	0	0	191	1900	unacceptable
41	0	0	187	4060	unacceptable
42	0	0	192	4060	unacceptable
43	350	0	158	0	acceptable
44	298	0	158	2860	good
45	298	0	158	3500	good
46	298	133	158	3870	good
47	245	0	158	2860	good
48	245	0	158	3500	good
49	245	263	158	3870	good
50	298	0	158	4400	good
51	298	0	158	6510	good
52	0	0	175	0	unacceptable
53	0	0	175	4060	unacceptable
54	0	0	175	1900	unacceptable
55	0	0	175	4060	unacceptable
56	0	0	175	1900	unacceptable

### 4.2. Classification

As was mentioned in Section 3.3, the chloride resistance of concrete depending on material composition can be searched using one of many software suites available on the market, and we decided to utilize the Weka workbench. The Weka workbench provides over one hundred algorithms supporting classification. They belong to different types, like: Bayesian classifiers, rule classifiers, tree classifiers or meta classifiers. In our research, we decided to determine the chloride resistance of concrete using the selected 20 algorithms belonging to three different types of algorithms. As a training set, all of the instances from the database (Table 6) were considered. The classification accuracy was evaluated using leave-one-out cross-validation. The obtained results are collected in Table 7.

**Table 7:** Results obtained for different classifiers from the Weka workbench.

Number	Classifier	Accuracy
Bayesian Classifiers		
1	BayesNet	66.07
2	ComplementNaiveBayes	62.50
3	NaiveBayes	73.21
Tree Classifiers		
4	BFTree	73.21
5	DecisionStump	73.21
6	FT	78.57
7	LADTree	82.14
**8**	**J48**	**89.29**
9	LMT	82.14
10	NBTree	78.57
11	REPTree	64.29
12	SimpleCart	71.43
Rule Classifiers		
13	ConjunctiveRule	71.43
14	DecisionTable	71.43
15	DTNB	80.36
16	JRip	62.50
17	NNge	76.79
18	OneR	71.43
19	PART	76.79
20	Ridor	66.07

**Figure 1:**
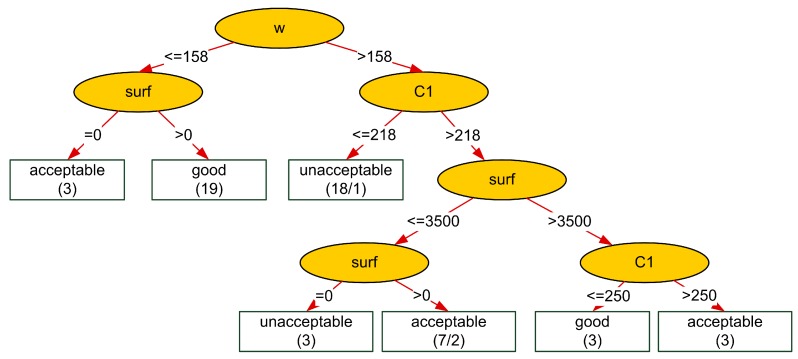
The decision tree for resistance to chloride penetration generated by the J48 algorithm.

The best accuracy equaling almost 90% was obtained using the J48 algorithm. The decision tree generated by the J48 algorithm is presented in Figure 1, where the first number in brackets denotes the number of examples from the training set covered by a selected leaf, and the second number, just after the sign “/”, indicates the number of incorrectly-classified instances (negative examples).

The obtained decision tree can be easily transformed into the following rules:
[resistance = good]
Rule 1 [w ≤158] and [surf >0]: *p* = 19, *n* = 0,
Rule 2 [w >158] and [surf >3500] and [218 < C1 ≤ 250]: *p* = 3, *n* = 0.

[resistance = acceptable]
Rule 1 [w ≤158] and [surf = 0]: *p* = 3, *n* = 0,
Rule 2 [w >158] and [C1 >218] and [0 < surf ≤ 3500]: *p* = 7, *n* = 2,
Rule 3 [w >158] and [C1 >250] and [surf >3500]: *p* = 3, *n* = 0.

[resistance = unacceptable]
Rule 1 [w >158] and [C1 ≤218]: *p* = 18, *n* = 1,
Rule 2 [w >158] and [C1 >218] and [surf = 0]: *p* = 3, *n* = 0,
where *p* denotes the number of positive examples covered by the rule (*i.e.*, the number of records from this class satisfying the rule) and *n* denotes the number of negative examples covered by the rule (*i.e.*, the number of records from the other classes satisfying the rule).

The obtained decision rules determine the conditions concretes have to fulfill to provide appropriate resistance against chloride penetration.

The good class characterizes:

- concretes with water content below 158 kg/m${}^{3}$ (w ≤ 158) where 15% or 30% of cement mass (C1 or C2) was replaced with high calcium fly ash (surf > 0),
- concretes with water content above 158 kg/m${}^{3}$ (w > 158) where 30% of cement C1 mass (218 < C1 ≤ 250) was replaced by high calcium fly ash S1 ground for 28 minutes or fly ash S3 ground for 20 minutes (surf > 3500).

The acceptable class characterizes:

- concretes without high calcium fly ash (surf = 0) with water content below 158 kg/m${}^{3}$,
- concretes with water content above 158 kg/m${}^{3}$ (w > 158) where 15% or 30% of cement C1 mass (C1 > 218) was replaced by unprocessed high calcium fly ash S1, S3 or S1 ground for 10 minutes (surf ≤ 3500),
- concretes with water content above 158 kg/m${}^{3}$ (w > 158) where 15% of cement C1 mass (C1 > 250) was replaced by high calcium fly ash S1 ground for 28 minutes or fly ash S3 ground for 20 minutes (surf > 3500),

The unacceptable class characterizes:

- concretes with water content above 158 kg/m${}^{3}$ (w > 158) and with a content of cement C1 below 218 kg/m${}^{3}$ (C1 ≤ 218), that is concretes containing cement C2 with or without high calcium fly ash, as well as concretes where 30% of cement C1 mass was replaced by unprocessed high calcium fly ash S3,
- concretes without high calcium fly ash (surf = 0) with water content above 158 kg/m${}^{3}$ (w > 158).

Using the leave-one-out method (*n* = 56), we obtained a classification accuracy equal 89.3%. The result obtained from a test set is often displayed as a two-dimensional confusion matrix with a row and a column for each class. Each matrix element shows the number of test examples for which the actual class is the row and the predicted class is the column. The sum of the numbers down the main diagonal divided by the total number of test examples determine the classification accuracy. The confusion matrix of the solved problem is determined in the form presented in Table 8.

**Table 8:** The confusion matrix for leave-one-out validation.

	good	acceptable	unacceptable
**good**	22	0	0
**acceptable**	0	9	3
**unacceptable**	0	3	19

Such a result can be considered satisfactory with respect to the limited number of records in the database.

## 5. Conclusions

The rules generated by algorithm J48 from the Weka workbench provided a means for the adequate classification of plain concretes and concretes modified with high calcium fly ash as materials of good, acceptable and unacceptable resistance to chloride penetration.

According to the generated rules, it is found that if the content of water in mixes is small enough (in investigated concretes, w ≤ 158 L/m${}^{3}$), then concretes modified with high calcium fly ash are qualified as materials of good resistance to chloride penetration, whereas concretes without high calcium fly ash are qualified as materials of acceptable resistance. For greater content of water (w > 158 L/m${}^{3}$), concretes using cement of low C${}_{3}$A with or without high calcium fly ash are characterized by unacceptable resistance to chloride penetration. However, when using cement of high C${}_{3}$A, the replacement 15% or 30% of cement mass by high calcium fly ash, particularly by ground fly ash, improves the resistance of concretes to chloride penetration.

It is found that both the specific surface of fly ash and the content of water and cement play a significant role in providing the required concrete resistance. The classifier was evaluated using the leave-one-out method. The obtained classification accuracy was equal to 89.3%. This value seems to be sufficient to acknowledge the correctness of the classifier. Due to a small number of tested specimens, the rules are applicable only to concrete mix compositions of similar binder content. Further tests are needed in order to enlarge the experimental database and to cover a broader range of concrete compositions.
